# BAG3: Nature’s Quintessential Multi-Functional Protein Functions as a Ubiquitous Intra-Cellular Glue

**DOI:** 10.3390/cells12060937

**Published:** 2023-03-19

**Authors:** Caitlyn M. Brenner, Muaaz Choudhary, Michael G. McCormick, David Cheung, Gavin P. Landesberg, Ju-Fang Wang, Jianliang Song, Thomas G. Martin, Joseph Y. Cheung, Hui-Qi Qu, Hakon Hakonarson, Arthur M. Feldman

**Affiliations:** 1 Department of Medicine, Division of Cardiology, Lewis Katz School of Medicine at Temple University, 3500 N. Broad Street, MERB 752, Philadelphia, PA 19140, USA; caitlyn.brenner@temple.edu (C.M.B.); muaaz.choudhary@temple.edu (M.C.);; 2 Center for Neurovirology and Gene Editing, Lewis Katz School of Medicine at Temple University, Philadelphia, PA 19140, USA; 3 Department of Molecular, Cellular and Developmental Biology, Colorado University School of Medicine, Aurora, CO 80045, USA; 4 Division of Renal Medicine, Brigham and Women’s Hospital, Harvard Medical School, Boston, MA 02115, USA; 5 Center for Applied Genomics, Children’s Hospital of Philadelphia, Philadelphia, PA 191104, USA; 6 Department of Pediatrics, The Perelman School of Medicine, University of Pennsylvania, Philadelphia, PA 191104, USA; 7 Division of Human Genetics and Division of Pulmonary Medicine, Children’s Hospital of Philadelphia, Philadelphia, PA 191104, USA; 8 Department of Pediatrics, Division of Human Genetics and Division of Pulmonary Medicine, The Perelman School of Medicine, University of Pennsylvania, Philadelphia, PA 191104, USA

**Keywords:** BAG3, heart, autophagy, apoptosis, excitation contraction coupling, mitochondria, cellular inhibitor of activation protein cIAP, mitochondrial uniporter

## Abstract

BAG3 is a 575 amino acid protein that is found throughout the animal kingdom and homologs have been identified in plants. The protein is expressed ubiquitously but is most prominent in cardiac muscle, skeletal muscle, the brain and in many cancers. We describe BAG3 as a quintessential multi-functional protein. It supports autophagy of both misfolded proteins and damaged organelles, inhibits apoptosis, maintains the homeostasis of the mitochondria, and facilitates excitation contraction coupling through the L-type calcium channel and the beta-adrenergic receptor. High levels of BAG3 are associated with insensitivity to chemotherapy in malignant cells whereas both loss of function and gain of function variants are associated with cardiomyopathy.

## 1. Introduction

Bcl2-associated athanogene 3 (BAG3) is a multi-functional protein involved in many biological processes that supports cellular homeostasis, cell structure, and cellular integrity. [1,2,3,4] *BAG3,* located on the long arm of chromosome 10, encodes for a 575 amino acid protein that is expressed in all mammalian tissues but is found most abundantly in the heart, skeletal muscle, central nervous system (CNS), and in many cancers [1]. A homolog of BAG3 has also been found in plants suggesting that BAG3 is a critical protein for the life of both plants and animals [5]. Consistent with its derivation from the Greek word meaning “anti-death”, this athanogene was first shown to play a significant role in inhibiting apoptosis or programmed cell death [6].

**Figure 1:**
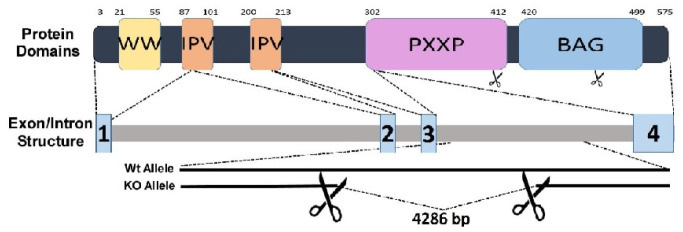

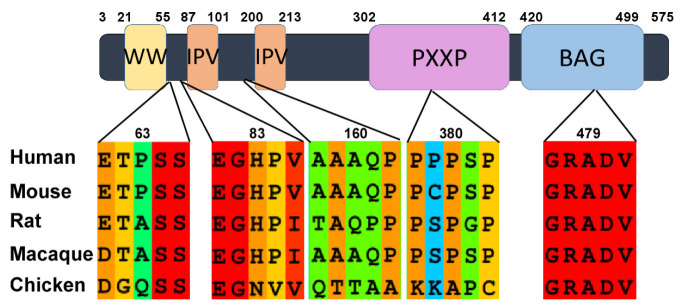
Structure of *BAG3*: BAG3 is a constitutively expressed, multifunctional, and ubiquitous protein that is most abundant in the heart, the skeletal muscle and the central nervous system. The top panel shows the structure of the BAG3 gene and the cleavage sites that result in the transcription of the four exons that are found in the mRNA that translates into the mature protein seen in the lower panel. The “WT” indicates the structure of the wild type gene or protein. The “KO” indicates the removal of a region of *BAG3* to create a knock-out phenotype in a mouse. The lower panel shows the locations of the amino acid changes that occur as a result of informative single nucleotide polymorphisms that change the amino acid translated from those genetic variants. To date, these novel variants have only been found in individuals of African ancestry and not in individuals of European ancestry. They do not alter the development of a dilated cardiomyopathy, but if present, they increase the risk of a patient who carries one of these variants either dying or developing worsening heart failure by almost two-fold.

BAG3 is one of six proteins in the BAG family. All of these proteins share a BAG domain that binds to the ATPase binding site on heat shock protein 70 (Hsp/Hsc 70), yet each appears to play a unique role in the cell [7] (Figure 1). All of the BAG family of proteins are constitutively expressed but are regulated independently. For example, BAG3 is the only stress-inducible member of the BAG-family whose expression can be regulated by heat, mechanical strain, and other pro-oxidative mechanisms [8]. BAG3 maintains the integrity of the cell by facilitating protein quality control (PQC) through lysosome-mediated autophagy and serves as a co-chaperone with both the large and the small Hsps to transport misfolded proteins to the proteosome and large protein fragments and diseased organelles (mitochondria) to autophagasome which, after binding with a lysosome, digest the proteinaceous materials by exposure to the lysosomal enzymes and the elevated acidity [9]. In the absence of BAG3, misfolded proteins and dysfunctional organelles accumulate in the cytoplasm leading to apoptosis (programmed cell death) or unprogrammed cell death (necrosis) with rupture of the cell membrane and the destruction of neighboring cells and tissues [10].

**Figure 2:**
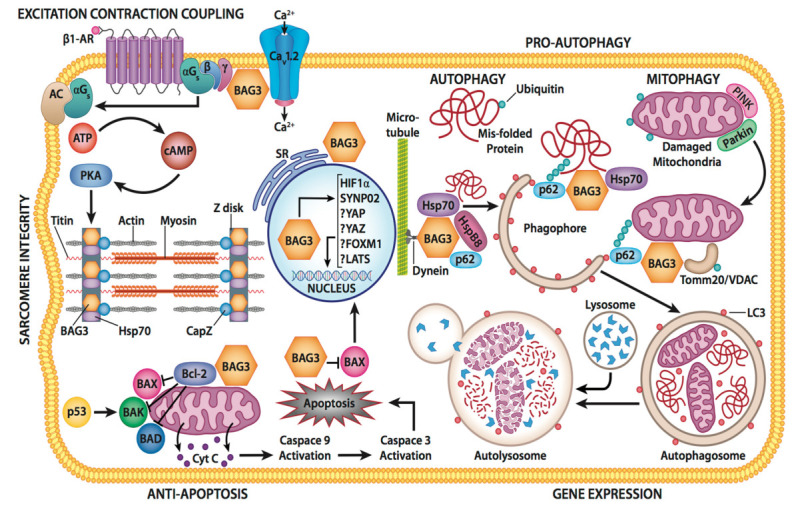
Diverse cellular roles of BAG3. The major pathways that require BAG3 for optimal functioning including excitation-contraction coupling, sarcomere integrity, Ca^2+^ homeostasis, autophagy, apoptosis, and gene expression. αG_s_—alpha subunit of the guanine nucleotide binding protein; β1-AR—beta1-adrrenergic receptor; AC—adenylyl cyclase; ATP—adenosine triphosphate; BAD—B-cell lymphoma-2-associated death; Bax—B-cell lymphoma-2-associated x protein; BAK—B-cell lymphoma-2 homologous promoter; antagonist/killer; Bcl-2—B-cell lymphoma-2; cAMP—cyclic adenosine monophosphate; Ca_v_-1.2 = L-type Ca^2+^ channel; Cyt C.—cytochrome C; FOX M1—forkhead box 14 M1 transcription factor; HF 1a—hypoxia-inducible factor 1 active transcription factor; LATS—serine/threonine protein kinase; LC3—microtubule-associated protein in 1A/B-light chain 3; p53—tumor suppressor protein; SR—sarcoplasmic reticulum; PKA—protein kinase a; ssYNPO2—protein coding gene synaptopodin 2; Tomm20 = mitochondrial import receptor subunit TOM20 homolog; VDAC—voltage dependent anion channel; YAP—yes-associated transcriptional regulator; YAZ—transcription factor.

BAG3 is ideally suited to the role of multi-tasking in view of its many protein-protein binding domains. However, unlike intracellular proteins that reside in specific niches of a cell such as receptors in the sarcolemma or contractile elements in the sarcomere, BAG3 is ubiquitous and appears to establish discrete intracellular micro-environments based on its specific responsibilities and locations (see Figure 2). For example, it couples the contractile elements to the Z disc in the cardiac sarcomere, couples the β-adrenergic receptor to the L-type Ca^2+^ channel in the sarcolemma, connects the dynein motor protein to the peri-nuclear aggresomes in the cytoplasm and co-chaperones the protein constituents of autophagy within the domain of the proteasome [11]. Recent work from our laboratories clearly shows that BAG3 also co-locates within the mitochondria, bound to TNF receptor-1 in the sarcolemma and the TOM proteins in the outer membrane of the mitochondria. Thus, rather than simply being a regulatory or even a structural protein, we posit that BAG3 serves as a universal glue that selectively localizes proteins to specific cellular domains as well as to partner proteins [12]. While not common, there are examples of this type of protein multi-tasking: the multi-functional protein 4.1R being a good example [13].

In this review we present an overview of the current literature surrounding BAG3 and its various roles in proteostasis, cardiac pathology, central nervous system disorders, cancer proliferation, and recently identified roles in maintaining the homeostasis of the mitochondria. We also discuss some potential pharmacological and non-pharmacologic interventions targeting BAG3 and associated proteins. BAG3 remains a protein that until recently has not been studied, as evidenced by the fact that its full array of cellular functions, and the mechanisms that control its expression and degradation, remain to be fully identified. However, it is becoming increasingly clear that that BAG3 plays a critical role in maintain normal function in the heart and in skeletal muscles and that its absence leads to a loss of function in these same organs. Although there are six members of the BAG family of proteins, this review will focus only on BAG3 because there are only nominal data regarding the roles that the other BAG family members play in the cell and in disease. We hope that this discussion will spark an interest in the research community and motivate new investigators to study the biology and regulation of this fascinating family of proteins which are clearly a member of the group of multi-functional proteins that participate actively in health and in disease.

## 2. Structure-Function Relationship

The BAG3 gene was first identified in 1999 by John Reed. He was working as an oncologic investigator who was using Bcl2 to screen a cDNA library in the hope of identifying a protein that could activate the Bcl2-dependent apoptosis pathway (intrinsic or mitochondrial dependent pathway of apoptosis) [14]. Early work studying BAG3 found that the protein was unique in that it had multiple protein binding sites throughout its relatively short 575 amino acids [15,16]. As seen in Figure 1, BAG3 has five well-defined protein-protein binding sites. Starting at the 3′ tail of the protein is the WW domain, which binds to proline-rich ligands such as LATS1/2, AMOTL1/2, guanine nucleotide exchange factor 2 (PDZGEF2), and synaptopodin-2 (SYNPO2). Such proteins play roles in tumor suppression, cancer development, and cell adhesion [17,18]. This is followed by two IPV (Ile-Pro-Val) motifs that mediate binding to other chaperone proteins, in particular the small heat shock proteins HspB6 and HspB8, which modulate various cellular functions [18,19]. Next is a proline-rich PXXP region which serves as an attachment point for the dynein motor pathway that carries cargo in a retrograde manner to the peri-nuclear aggresomes for eventual transport to the proteome [20]. The PXXP domain has also been implicated in cellular protection against hypoxia-reoxygenation stress in cardiomyocytes via its binding to Hsp70 [21]. The BAG domain binds to the ATPase binding site on Hsp70, thereby facilitating the ability of BAG3 to serve as a co-chaperone and also binds to Bcl2, a member of the 25-member family of Bcl-2-like proteins that can either activate or antagonize (Bcl-2) the mitochondrial-dependent (or intrinsic) activation of apoptosis.

It should be noted that despite our growing understanding of the role that BAG3 plays in human disease and the beginning of an understanding of the relationships between BAG3 genotype and the resultant mouse or human phenotype, we do not know the 3-dimensional structure of the mammalian protein as the relevant data have not been deposited in the Protein Data Bank (PDB) database for either the protein itself or for any of its domains. Nonetheless, some information is available from the crystallization and structural analogous BAG family members that are found in plants [22]. In the four members of the BAG family found in the *Arabidopsis thaliana* plant in which there are four members of the BAG, the domain organization is similar to that seen in mammals. The BAG proteins adopt a structure consisting of three short parallel α-helices similar to some mammalian BAG proteins. Interesting information about the structure of BAG3 can also be gleaned from the recent AlphaFold database that predict that the protein is rich in intrinsically disordered protein domains (https://alphafold.ebi.ac.uk/entry/095817). This artificial intelligence database raises the question of whether BAG3 has structural elements that could serve as potential pharmacological targets; however, as discussed later in this review, several pharmacologic agents have been shown to increase BAG3 levels; however, the mechanism by which they alter BAG3 levels remains to be determined. The database is also interesting in that the majority of potential functional sites are associated with myofibrillar disease—a likely outcome of genetic variants in the P209 domain but not seen with the more common deletions, truncations, and non-sense variants that result in haplo-insufficiency.

## 3. Cellular Function of BAG3

### 3.1. Protein Quality Control

One of the two primary responsibilities of BAG3 in the cell is to maintain cellular homeostasis by regulating autophagy (Figure 2). Under physiologic conditions, protein turnover/degradation is mediated by the protein BAG1 and the heat shock protein 70 (Hsp70) that carry the protein to a proteasome for degradation. When the cell is stressed by environmental changes or pathological conditions, such as those occurring during physiologic stress or disease, the demand for protein degradation increases and proteasomal degradation is inadequate. Furthermore, the proteosome has strict limits on the size of the protein that it can accommodate and is not able to rid the cell or damaged organelles and in particular damaged mitochondria. The cell accommodates for any increased need to rid the cell of misfolded proteins or organelles by switching from a BAG1-dominant protein clearance to the more efficient and higher volume BAG3-mediated selective autophagy pathway. While BAG3 serves as a co-chaperone with Hsp70 under both basal and stress conditions, BAG3 is crucial for the regulation and degradation of protein aggregates that result from stress as well as from both age and disease processes [3]. The increase in the BAG3/BAG1 ratio lowers the efficiency of the ubiquitin-proteosome, while also stimulating macroautophagy [19]. During the ubiquitin proteasome system (UPS) pathway, the BAG1/Hsp70 interaction is involved in proteasomal delivery of Hsp70 client proteins to the proteasome [3]. It has been suggested that when the BAG1 to BAG3 switch occurs, BAG3 prevents Hsp70-mediated protein degradation. This is thought to be a result of binding competition between BAG1 and BAG3 onto Hsp70. As a result, during stress, the BAG3/BAG1 ratio increases allowing BAG3 to outcompete BAG1 for Hsp70 binding [19]. The resulting interference with BAG1/Hsp70-client protein complex formation results in less misfolded protein substrates being delivered to the proteasome. Instead, misfolded proteins bind to BAG3 along with increase in the activity of the autophagy lysosomal pathway whereby misfolded proteins are digested by lysosomal proteins.

During macro-autophagy, large protein aggregates, pathogens, and damaged organelles, such as mitochondrion and other BAG3-bound cargo proteins, bind with p62, a ubiquitin-binding protein, and with the microtubule-associated protein 1A/1B-light chain 3 (LC3) to form a phagophore (Figure 2). When the complex is fully loaded, the two open ends fuse to form an autophagosome. A lysosome then fuses with the autophagosome which allows lysosomal enzymes to flow into the autophagosome where they proceed to digest the proteins into their individual amino acids. The amino acids are then released and used to produce new proteins [3,19]. This specific BAG3 pathway is also referred to as chaperone-assisted selective autophagy (CASA) [23]. CASA is especially relevant to skeletal and cardiac muscle as the CASA machinery is localized to the Z-disc [24]. CASA knockdown leads to disintegration of the Z-disc and pathological changes in the skeletal and cardiac muscle. In addition to pathological states, an increase in the BAG3/BAG1 ratio has also been seen during the normal aging process [19]. This increase in BAG3 could be a protective mechanism as proteins frequently oxidize and aggregate as organisms age [19].

### 3.2. BAG3 and Autophagy

BAG3 synthesis can be activated in both physiological and pathological situations. Its expression is induced by stress, proteasome inhibitors, 50 Hz electromagnetic fields, and aging [5,20,25,26,27]. BAG3 is also regulated by a diverse array of transcription factor, the most important being the large and small heat shock proteins, heat shock factor 1 (HSF1), as well as other transcription factors such as WT1 and Egr1 [19]. When BAG3 binds to HSF1 via its BAG domain, it facilitates shuttling of HSF1 from the cytosol to the nucleus where HSF1 can activate expression of HSF1-dependent genes, but HSF1 also interacts with two elements in BAG3′s promoter to increase its expression in an NF-kB-dependent manner [19,28]. BAG3 is also sensitive to an additional group of transcription factors including the androgen-regulated protein androgen-induced b-ZIP (AlbZIP) [29], fibroblast growth factor 2 (FGF-2) [30], and early growth response proteins. BAG3 is also expressed in endothelial cells, where its absence leads to decreased angiogenesis. It can sequester the LATS1/2 and AMOTL1/2 kinases, which prevents them from binding to their transcription coactivators YEP and TAZ, ultimately preventing them from entering the nucleus [19,31,32]. The WW domain of BAG3 has also been shown to bind proline-rich motifs of the tuberous sclerosis protein TSC1. This protein can form a complex with TSC2 to inhibit the mammalian target of rapamycin complex (mTOR), which regulates translation. Ultimately, this causes an increase in translational efficiency. The BAG domain of the BAG3/Hsp70 complex also couples to the ubiquitin ligase carboxy terminal of the Hsp70/Hsp90 interacting protein (CHIP) [33]. This interaction allows misfolded proteins to be sequestered into autophagosomes for degradation [34]. Additionally, BAG3 catalyzes substrate transfer from Hsp70 to the dynein motor complex. Protein aggregates bind to the BAG3/HspB8/Hsp70 complex, and BAG3 then interacts with the 14-3-3γ protein to mediate the association between the BAG3/HspB8/Hsp70 complex and dynein [4]. Finally, BAG3 was found to colocalize to the Z-disc in response to stress, and interacted with Hsp70 and HspB8. This complex is known to engage in the CASA pathway to transport misfolded proteins to the autophagosome for degradation. In these mice with heart failure with reduced ejection fraction (HFrEF), the decrease in BAG3 directly correlated with a decline in F_max_. However, the maximal force generated by the myofilament (F_max_) and sarcomere protein turnover were restored upon treatment with adeno-associated vector (AAV) gene therapy [35].

The 14-3-3γ protein serves as a molecular link between BAG3 and dynein. BAG3′s interaction with dynein has been shown to be mediated by its PXXP domain, which serves to anchor the proximal end of the dynein motor pathway. Meanwhile the 14-3-3γ protein binds to the phosphoserine-containing 14-3-3 binding motifs of BAG3 and to the dynein-intermediate chain [18]. A mutation of serine at position 136 or 173 in the phosphoserine-containing 14-3-3 binding motif of BAG3 abolishes this interaction [18,36].

Any discussion of the role of the HSPB8-BAG3-HSP70 complex in facilitating autophagy in the heart or the brain is not complete without an introduction to a new area of great interest and potential therapeutic use: stress granules [37]—stress granules (SGs) are membrane-less cytosolic assemblies that form in response to stress and have been associated with diseases as diverse as hearing loss, neurodegenerative disease, and heart failure [38]. That BAG3 might play a role with the development and the maintenance of functional stress granules was supported by the finding that the HSPB8-BAG3_HSP70 chaperone complex maintains the integrity of the stress granule and prevents the accumulation of dysfunctional complexes in stress granules during stress [39]. Cherkasov et al. showed that genetic depletion of the HSP-BAG3-HSP6-8d complex delayed stress granule dissolution in mammalian cells and others have shown that the small heart shock protein HSPB8 and 6 are important for maintaining the integrity of the complex thereby ensuring the functionality of the stress granules and restoring appropriate levels of proteostasis [40]. Importantly, Ganassi et al. recently confirmed these findings using cells from patients with amyotrophic lateral sclerosis thereby supporting the importance of the hspb8-BAG3-HSP70 chaperone complex in maintaining cellular homeostasis, although the applicability to heart failure remains to be identified [41].

### 3.3. Apoptosis and Oxidative Stress

As an anti-apoptotic protein, BAG3 can inhibit caspase activation. It does so by influencing the two mechanisms of apoptosis: the intrinsic (mitochondrial dependent) pathway and the extrinsic (receptor mediated programmed cell death) pathway (Figure 2). In the intrinsic pathway, BAG3 binds to Bcl2, one of 24 members of the Bcl2 family of proteins. These proteins can either increase or decrease activation of the mitochondrial release of cytochrome c and AP1 from the mitochondria with subsequent activation of caspase 9 and caspase 3. However, Bcl2 is a primary inhibitor of the pathway which cooperates with BAG3 in inhibiting the release of cytochrome c from the mitochondria with the subsequent inhibition of caspase activation. We have recently shown that BAG3 also binds to the cell inhibitor of apoptosis protein1/2 (cIAP1/2), which stabilizes the attachment of the inhibitor protein to a caspase and therefore prevents it from being cleaved [12]. In addition, BAG3 may also bind to a cIAP that is attached to the TNFR1 receptor and thus might also inhibit activation of TNFα signaling [12]. Thus, the role of BAG3 in the inhibition of release of cytochrome c and in the sequestration of the pro-caspases 8 and 9 and its effect on oxidative stress-associated endothelial biology remains controversial [42,43]. It is important to note that there is evidence for BAG3′s role in response to oxidative stress-associated endothelial damage as endothelial specific BAG3 knockout mice demonstrated an increase in oxidative stress-associated endothelial damage and vascular remodeling [44], whereas BAG3 overexpression significantly decreased the damage associated with this process.

One of the most interesting recent observations in the area of BAG3 and the heart was the finding that in addition to BAG3, poly(ADP-ribose) polymerase 1 (PARP1) is also involved in oxidative stress-induced endothelial damage [44]. Activation of PARP1 promotes the consumption of NAD, signaling a low energy state, and subsequently shuts down high energy consuming processes [44]. BAG3 is able to bind to the BRCT domain of PARP1 which in turn promotes its degradation. BAG3 binding also promotes the ubiquitination of PARP1 by the E3 ubiquitin ligase WWP2. WWP2 is also responsible for mediating pathological cardiac fibrosis via activation of TGFβ/Smad [45]. Under normal physiologic conditions, BAG3 is also a substrate of both the acetyltransferase CREB-binding protein (CBP) as well as the deacetylase sirtuin 2 (SIRT2). These two enzymes are responsible for acetylating and deacetylating BAG3 at its K431 residue, respectively [44]. Importantly, it is the deacetylated form of BAG3 that has been shown to promote the ubiquitination of PARP1 [44]. We recently found that PARP is significantly elevated in hearts from mice with a single allele knock-out of BAG3 even before the onset of cardiac remodeling and diminished left ventricular function [12]. Consistent with the studies in mice, PARP is also elevated over two-fold in the ventricular myocardium of human tissue obtained from hearts of patients with non-ischemic heart failure obtained at the time of cardiac transplantation, thus its role in the biology of heart failure and fibrosis requires additional evaluation. In fact, PARP might be an important regulatory protein for BAG3-mediated events.

### 3.4. BAG3 and the Sarcomere

A constant feature of a failing human heart is a reduction in the force-generating capacity (Fmax) of the myofilaments, the primary component of the sarcomere that are responsible for maintaining the micro and macro contracting capabilities of the heart [35]. No area of mammals or humans is better able to illustrate the multiplicity of effects of BAG3 and to support our contention that its primary role is to serve as a ubiquitous glue, than its role in the sarcomere. In the sarcomere, BAG3 not only forms the underpinning for the cellular and molecular mechanisms that hold the elements of the contractile fibers together, but it also modulates the levels of functional filamin by making sure that the protein is properly folded and by stimulating the de novo synthesis of filamin when its levels are reduced [35]. This activation of de novo synthesis occurs when filamin is degenerated via YAPZ-activated autophagy [19]. However, BAG3’s role in the sarcomere appears to be even more complex than that of a simple glue [46]. For example, it is also involved in actin folding through its interaction with the cytosolic chaperonin TL-complex protein ring complex (TriC) [47], operates by chaperone-assisted selective autophagy to remove misfolded proteins (CASA), supports activated protein refolding, and maintains both the function and the localization of the sarcomeric CASA complex. In addition, BAG3 can interact with several proteins that play a role in stabilizing the myofibrillar Z-disk.

Myogenin is another protein that is important for muscle homeostasis and the terminal phases of myogenesis [46]. As such, altering its levels can affect primary human cardiomyocytes. De Marco et al. found that BAG3 levels were higher in differentiated H9c2, rat-derived myocytes in comparison to undifferentiated myoblasts. BAG3 levels increased during myoblast differentiation which suggests its preferential role in differentiated myocytes [48]. This result supported the finding that homozygous deletion of *BAG3* resulted in a fulminant myopathy and early lethality, yet no obvious abnormalities could be found in the fetus [49] correct.

### 3.5. BAG3 and Insulin Release

BAG3 also appears to play a role in insulin release via two mechanisms [19]. Insulin is released from vesicles when SNARE proteins form a complex to initiate the fusion of the vesicle and cell wall. Importantly, F-actin forms a barrier along the plasma membrane that prevents this fusion [50]. The first mechanism of BAG3 is its ability to affect the polymerization state of F-actin, in turn affecting the ability of vesicles to secrete insulin. When BAG3 is silenced, this results in an increase in insulin secretion. Secondly, BAG3 binds to SNAP-25 and syntaxin-1, which are component proteins of the SNARE complex, thereby inhibiting their ability to interact with one another [50]. Without their interaction, the SNARE complex is not formed and fusion with the cell membrane cannot occur. Upon glucose stimulation, BAG3 is phosphorylated by focal adhesion kinase (FAK) [34]. This phosphorylated form of BAG3 causes it to dissociate from SNAP-25, thereby regulating insulin release in response to glucose [50]. BAG3 has been shown to have high expression in pancreatic islet cells and has been found, via co-immunostaining, to colocalize with insulin-containing granules [19,50].

### 3.6. Cell Cycle

To ensure cell survival, cell cycle progression must be closely monitored. BAG3 plays a role in this process by forming a complex with DUSP6 and phospho ERK (pERK). This complex is involved in cell cycle progression. Upon BAG3 silencing, there is an increase in G1 block. This is a result of increased levels of the Cdk inhibitors p15 and p21 [51].

### 3.7. BAG3 and Cell-Cell Junctions

The functions of BAG3 have also been linked to the protein connexin 43 (Cx43), which plays an important role in cell–cell communication in excitable membranes including the heart [52]. Cx43 is found in gap junctions and is critical for both metabolic and electrical coupling of cardiomyocytes as well as other cells [52]. In fact, a loss of function in the gap junctions has been associated with HFrEF as well as lethal arrhythmias [18,53]. BAG3 has also been shown to regulate the physiologic maintenance and pathologic degradation of Cx43. When alkaline phosphatase (ALP) levels are diminished, degradation of Cx43 becomes dysregulated. ALP can be impaired either by inhibiting lysosomal activity or by suppressing BAG3 levels. When lysosomal activity is inhibited in cardiomyocytes, there is an accumulation of Cx43 aggregates, and when BAG3 is knocked down, Cx43 levels also decrease due to diminished protein turnover [52,54]. BAG3 colocalizes with α-tubulin in neonatal rat ventricular cardiomyocytes, and Cx43 was found to be surrounded by a tubulin network in the perinuclear area. When this tubulin network is depolymerized, Cx43 localization to the plasma membrane is inhibited. Additionally, there was an accumulation of non-phosphorylated Cx43 in the cytoplasm, which is associated with arrhythmogenesis and HF [49,55]. BAG3 aggregates were also observed in cardiomyocytes treated with depolymerizing agents. This suggests that both BAG3 and tubulin are important proteins for maintaining the integrity of gap junctions and regulating excitation–contraction coupling.

### 3.8. BAG3 and Proteasome Inhibitors

Proteasome inhibitors have been used in the treatment of cancer, in particular pancreatic tumors and lymphoma [56]. Exposure to the proteasome inhibitor, bortezomib, has some effect on the tumor but also increases the levels of tissue BAG3 and its related chaperones. It has been suggested that this could be a cellular response to the need for an alternative degradative pathway; however, that is unlikely as a similar proteasome inhibitor, carfilzomib, has a similar effect on the proteosome with a greater incidence of cardiotoxicity as compared to bortezomib [57]. That bortezomib’s effects on increasing BAG3 are of clinical relevance (i.e., increasing levels of BAG3) is supported by the finding that cardiotoxicity is substantially greater with carfilzomib then it is with bortezomib—suggesting that carfilzomib inhibits the proteasome, thereby effectively blocking the ability of the cell to rid itself of the debris associated with increase replication and growth, whereas bortezomib also inhibits the proteasome to a similar degree as does carfilzomib, but also increases the cellular levels of BAG3 with an accompanying increase in BAG3-mediated autophagy. Not surprisingly, the administration of carfilzomib has a greater risk of cardiotoxicity than does bortezomib—but with improved tumor destruction and better outcomes for patients with cancer [58].

### 3.9. Effects of BAG3 Depletion on the Proteosome the Inflammasome and the Metabolome

Traditionally, proteomic evaluation occurs after the disease phenotype has evolved and therefore the adverse biology that leads to organ dysfunction may be obscured. To obviate the effects of common HF-related abnormalities on cardiac biology we performed proteomic evaluation on samples of mouse myocardium obtained from mice in which a single allele of BAG3 was deleted, and compared them with proteomic analysis performed on non-transgenic littermate controls. Proteomic and confirmational Western blot analysis showed a decrease in BAG3 levels of approximately 50% as was expected. We also found that BAG3 plays a role in modifying the extrinsic pathways of apoptosis and in regulating inflammation through activation of the cardiac inflammasome and in particular the TNFR1 signaling cascade [12]. BAG3 played an equally important role in supporting transport of Ca^2+^ into and out of the mitochondria. This transport of Ca^2+^ maintains the mitochondrial membrane potential that is required for Ca^2+^ flux—the biological event that provides the energy required by the enzymes of the tri-carboxylic acid (TCA) cycle.

The cellular machinery that enables BAG3 to remove inextricably damaged or diseased organelles and cells from tissues without the collateral damage that occurs when cells die suddenly is complex and involves multiple regulatory pathways. When cell death is programmed as in apoptosis, the cell membrane remains intact and internal enzymes and toxic materials are degraded prior to the end of the cell as a functioning organelle. As noted by Haudek et al. over a decade ago, two canonical pathways regulate apoptosis: the type I or the type II pathway [59]. In type 1 (mitochondrial independent of extrinsic), the pathway that will lead to cell death begins with activation of a death domain receptor (commonly tumor necrosis factor receptor-1:TNFR-1) and ends with activation of the executioner caspases 7 and 3. In contrast, in the type II (mitochondrial-dependent) pathway, apoptosis is activated by the BAG3-dependent release of pro-apoptotic signals from the mitochondria including cytochrome c and endonuclease g with the subsequent activation of caspases 9 and 3.

Haudek proposed that apoptotic cell death does not necessarily occur as a direct result of the activation of the cell death pathways but is due instead to sustained TNF signaling that leads to cell death after one or more anti-apoptotic proteins become depleted. Over-expression of Bcl-2 was able to partially block this pathway. We recently reported that the absence of a full complement of the strongly anti-apoptotic BAG3 protein resulted in a very similar phenotype in the BAG3 haplo-insufficiency model with an increase in total caspase 3 activity and caspase 8 activity. What is unique to the haplo-insufficiency BAG3 model is an increase in the levels of TNF and an adverse change (decrease) in the mitochondria membrane potential independent of changes in the amount of cellular inhibitors of apoptosis (cIAP). Greater clarity might be gained if cIAP is found to bind to both the TNFR1 receptor and to BAG3.

**Figure 3:**
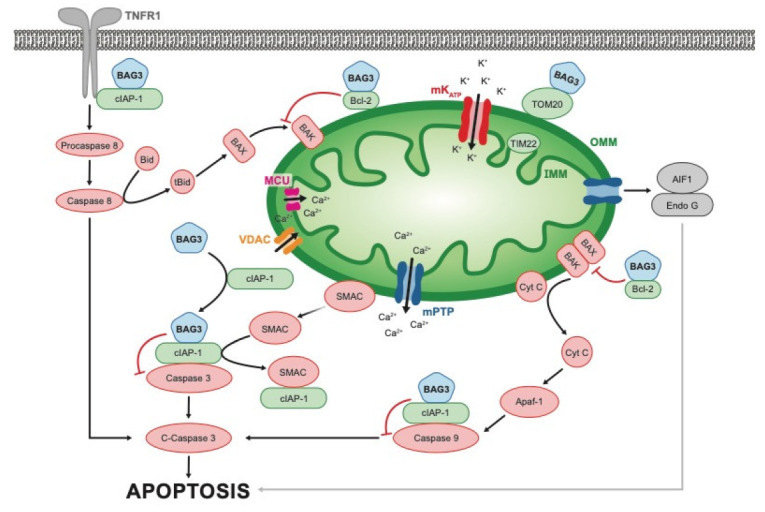
Canonical and non-canonical pathways for mitochondrial regulation of apoptosis and inflammation: abbreviations: cIAP-1/2—cellular inhibitor of apoptosis; IMM internal mitochondrial membrane; IMM—internal mitochondrial membrane; OMM—outer mitochondrial membrane; Cyt C—cytochrome C; MCU—mitochondrial calcium uniporter; SMAC (*DIABLO)* second mitochondria-derived activator of caspase; TOM20—mitochondrial import receptor subunit encoded by the *TOMM20* gene; AIF-1—allograft inflammatory factor 1 (ionized calcium-binding adapter molecule 1, encoded by the *AIF1* gene; mPTP—mitochondrial permeability transition pore; TNFR-1—tumor necrosis factor receptor-1; TIM22—translocase of the inner mitochondrial membrane; ATP—adenosine triphosphate.

We have recently shown in young mice with BAG3 haplo-insufficiency but in whom the ejection fraction is normal and cardiac remodeling has not begun, that the loss of one allele of BAG3 and the resulting decrease in BAG3 levels by 50% was associated with an increase in caspase 3 but not a change in the ratio of cleaved caspase 3/total caspase 3 [12]. Unexpectedly, we found an increase in caspase 8 and cleaved caspase 8 and an increase in the ratio of cleaved caspase 8 to total caspase 8 which is associated with an increase in apoptosis (Figure 3). Once activated, caspase 8 is responsible for apoptosis induced by the death receptors—a biological change involving TNFR1 and DR3. Consistent with this finding, deletion of caspase 8 and RIPK3 prevent aberrant cell death, reduced the amount of inflammation and prolonged mouse survival [60]. Caspase 8 has also been studied in cancer [60,61,62] where cIAPs and their antagonists regulate spontaneous and TNF-induced proinflammatory cytokine and chemokine production [63]. In view of our recent studies that unequivocally confirmed that BAG3 immuno-precipitates with cIAP, and cIAP is known to couple with TNFR1 at the junction of TRAF2/TRAF5, LUBAC, and RIPK1, we suggested that the presence or absence of BAG3 might have a critical role in TNF signaling in the heart and in the inflammasome of myocardial cells [64].

The mitochondrial Ca^2+^ (mCa^2+)^ uniporter, the major pathway for Ca^2+^ uptake by the mitochondria, plays an important role in the heart as it is responsible for ATP production by the tri-carboxylic acid cycle and in particular the activity of the Ca^2+^-regulated enzymes of the tri-carboxylic acid cycle [65]. In fact, it was recently reported that an increase in BAG3 is associated with an increase in the Ca^2+^-dependent tri-carboxylic enzymes including pyruvate dehydrogenase, alpha ketoglutarate dehydrogenase, and isocitrate dehydrogenase [66]. Supra-normal levels of mCa^2+^ result in an increased workload, thereby increasing cell stress, whereas the opposite occurs when mCa^2+^ levels are low and stress is minimized [67]. The mitochondrial Ca^2+^ mitochondrial uniporter (MCU) is the major pathway for Ca^2+^ uptake by the mitochondria, whereas the mitochondrial permeability transition pore is the site through which excess Ca^2+^ is lost. We also found a decrease in MCU level and activity in BAG3^+/−^ hearts and propose that this contributes to the HFrEF phenotype seen in older BAG3 haplo-insufficient mice [12]; however, the biology of mCa^2+^ is complex and not without controversy [68,69], In fact, the mechanisms responsible for the molecular and cellular biology of the mitochondria, particularly how they relate to Ca^2+^ homeostasis, have also been debated [67,70,71]. In view of the controversy, our observation that restoration of normal levels of BAG3 improves mitochondrial Ca^2+^ uptake and by extension enhances cellular bioenergetics, thereby providing benefit to the cell, requires additional study.

### 3.10. BAG3 Secretion/Excretion/Leak

Pancreatic ductal adenocarcinoma (PDAC) has been shown to produce large quantities of BAG3s and the levels of BAG3 are inversely related to survival [72]. In fact, the appearance of increased quantities of BAG3 in the peripheral blood serves as a marker for PDAC [73]. Once secreted, BAG3 binds to macrophages via the interferon-induced transmembrane protein 2 (IFITM-2), a cell-surface receptor resulting in macrophage activation and the secretion of PDAC supporting factors [72,73]. Further, treatment with a murine anti-bag3 antibody resulted in lower numbers of stromal macrophages suggesting that the antibody can prevent macrophage activation and infiltration of the tumor stroma, as well as reduced growth and metastasis [74]. Basile et al. highlighted the potential of this therapy by demonstrating specific cellular localization and reduced growth of pancreatic cancer cell xenografts when exposed to an anti-BAG3 humanized antibody [75].

### 3.11. Cell Metabolism

Tumor cells use aerobic glycolysis, a phenomenon known as the Warburg effect, to keep up with their energy demands as they grow and proliferate, and it has been shown that BAG3 shifts malignant cells to aerobic glycolysis [1]. BAG3 overexpression promoted glycolysis in PDAC cells, while BAG3 knockdown led to suppressed glycolysis in PDAC cells [76]. Mechanistically, BAG3 stabilizes hexokinase-2 mRNA which leads to increased hexokinase-2 (HK2) expression helping to promote aerobic glycolysis in pancreatic cancer cells, while BAG3 knockdown destabilizes HK2 [76]. Additionally, glutaminase (GLS), an enzyme that converts glutamine to glutamate, is involved in cancer cell metabolism, growth, and proliferation and in certain cancers is regulated by BAG3 [77]. Glutaminolysis, which begins with GLS, provides proliferating cells with a nitrogen, sulfur, and carbon skeleton for macromolecule biosynthesis and maintains redox balance, and a variety of other roles that enable cancer growth and survival [78]. Zhao et al. demonstrated that BAG3 stimulated autophagy through the stabilization of GLS and thus increased glutaminolysis [78]; a study supported more recent data showing that GLS is required for tumorigenesis [79]. Perhaps the most important role that BAG3 plays in cell metabolism is to maintain homeostasis of the mitochondria. In a recent study, we found that mitochondria isolated from adult mouse hearts in which one allele of BAG3 had been knocked out demonstrated a less-negative membrane potential and a decrease in the M1 protein of the mitochondrial uniporter [12] (Figure 3).

### 3.12. Angiogenesis

Another role for BAG3 in cancer is its involvement in angiogenesis as neo-angiogenesis is critical for the growth and progression of cancer cells. BAG3 downregulation has been shown to result in decreased angiogenesis, through a mechanism involving ERK phosphorylation [51]. BAG3 is required for the interaction between ERK and DUSP6, and ensures that phosphorylated ERK does not reach levels sufficient to promote expression of p21 and p15 and cell cycle arrest in G1. It has also been shown that BAG3 can induce vascular endothelial growth factor (VEGF) in cancer cells, thus supporting angiogenesis, and knockdown of BAG3 resulted in reduced VEGF [80]. Importantly, BAG3 silencing resulted in reduced tumor neo-angiogenesis [51]. In studies noted earlier in mice with a naturally occurring loss of function of BAG3, preliminary studies suggested that the injection of an AAV vector driving the expression of BAG3 was associated not just with enhanced muscle survival and ambulation but also with increased angiogenesis. The associated biology remains to be worked out.

## 4. BAG3 in Heart and Skeletal Muscle

### 4.1. BAG3 Genetic Variants and Dilated Cardiomyopathy

In 2006, Homma first reported that mice with a homozygous deletion in *BAG3* develop non-inflammatory myofibrillar degeneration and subsequent death by four weeks of age and that mice with heterozygous deletion survived [49]. This finding diminished interest in BAG3 as a potential therapeutic target. However, interest quickly re-emerged when Selcen and colleagues reported that an informative single nucleotide substitution that changed the amino acid at position 209 from a proline to a leucine (P209L) led to a phenotype that was characterized by profound skeletal muscle weakness, giant axon disease and mild cardiac hypertrophy leading to eventual death in the early teens due to respiratory failure [81]. The P209L variant is now recognized as being a dominant gain of function mutation, the pathology of which leads to extensive stalling of the Hsp70 networks [8]. As pointed out by Adriaenssens, even a single nucleotide substitution can have significant consequences in a multi-functional protein such as BAG3. The same proline codon (P209) with a different amino acid transcribed from the second homologous chromosome could result in a dilated cardiomyopathy or Charcot–Marie-tooth type 2 neuropathy through a similar toxic gain-of-function that leads to the accumulation of the variants in the form of insoluble HDAC6- and vimentin-positive aggresomes [82]. The accumulation of these insoluble aggresomes caused the relocation of other chaperones including HSPB8 and Hsp70 together with wild-type BAG3. Although the BAG3 P209L protein is functional, it is trapped leading to increased aggregation-proneness and ubiquitination of client proteins with the long-term effect of inefficient clearance of misfolded proteins and other cellular debris [83]. In addition, the P209Q mutation is associated with adult-onset mid-sensorimotor polyneuropathy and an absence of giant axons [84] and P209S causes myopathy and neuropathy [8].

That genetic variants in BAG3 could result in more traditional heart failure as evidenced by cardiac dilatation and diminished ejection fractions was first noted by Ellinor and colleagues who identified a locus on chromosome 10 that was informative for the development of HFrEF in a large cohort of patients with dilated cardiomyopathy [85]. A series of subsequent studies, by both ourselves and others, identified specific genetic variants in BAG3, the majority of which were either deletions or truncations [86,87,88,89]. In 2014, we found that mice with cardiac-specific heterozygous KO of BAG3 could survive up to 10 weeks, whereas mice with a cardiac-specific heterozygous deletion mutations could survive to between 20 and 40 weeks of age [90]. Thus, genetic mice provided a useful model in which to study BAG3 biology. Most interesting was the finding that BAG3 levels are reduced by 50% in patients with end-stage heart failure, independent of a genetic variant [89]. The importance of BAG3 to the life of each cell is documented by the fact that to date, no living species has been identified with a homozygous deletion. However, it was a study from a European consortium of centers who studied over 100 patients with familial dilated cardiomyopathy that gave us the best view into the human disease [91]. Their finding were notable for a relative early penetrance of the disease, a more rapid progression of disease from the earliest symptoms to substantial disease, a substantial preponderance of deaths from worsening heart failure (as versus sudden cardiac death), and a better outcome in women, a finding that was supported by subsequent studies in mice [35,92].

In contrast to potential evolutionary advantage and positive selection of heart protective BAG3 variation, damaging BAG3 variation tends to rarely be related to glioblastoma cells. In glioblastoma, BAG3 binds bax in the cytosol which prevents bax translocation to the mitochondria and thus inhibiting apoptosis. Whole exome or whole genome sequencing, the importance of BAG3 variants in the development of HFrEF, has been clarified. Using a sequencing panel to evaluate the burden of rare variants in 56 putative dilated cardiomyopathy (DCM)-associated genes in 1040 patients with DCM and 912 healthy controls, Mazzarotto et al. found that truncating variants in TTN and DSP were associated with DCM in all comparisons, whereas variants in MYH7, LMNA, BAG3, TNNT2, TNNC1, PLN, ACTC1, NEXN, TPM1, and VCL were significantly enriched in specific patient subsets, which for BAG3 included a diagnostic referral DCM cohort but not a primary care outpatient clinic cohort [92]. In a group of similarly large GWAS studies, the BAG3 locus was associated with dilated cardiomyopathy [93]. Most recently, we reported that all of the known pathogenic/likely pathogenic variants affect at least one of three protein functional domains: the WW domain, the second IPV (Il-Pro-Val) domain, or the BAG domain; whereas none of the missense pathogenic/likely pathogenic variants affect the proline-rich repeat (PXXP) domain [7]. A common variant, p.Cys151Arg, associated with reduced susceptibility to dilated cardiomyopathy, demonstrated a significant difference in allele frequencies among diverse human populations, suggesting evolutionary selective pressure. In addition, 5 eQTL SNPs are also associated with primary cardiomyopathies by FinnGEn Biobank genome-wide association study.

In 2020, we identified three novel BAG3 variants among an African American population with HFrEF, but these variants were not found in a population of European ancestry with HFrEF. These were nonsynonymous single nucleotide variants in P380S, A479V, and P63A [94,95]. Subsequently we found that the P63A and the P380S were on the same strand and in the same patients so the correct abbreviations would be BAG3 ^63/380^. The possession of any one of these variants was associated with a worse cardiac outcome [94]. When haplo-insufficient mice were compared with mice expressing one WT and one mutant allele (either P380S, A479V, or P63A/P380S), the mice harboring one of the mutant alleles had a comparable decrease in excitation-contraction coupling in response to isoproterenol when compared to haplo-insufficient mice. This suggests that, in mice, these mutations behave like loss-of-function mutations. The locations of these mutations implicate the WW (P63A), PXXP (P380S), and BAG (A479V) domains. However, it was found that only the BAG domain is required for proper β-adrenergic responsiveness. Circulating levels of catecholamines are elevated in patients with HF, when an increase in muscle function or cardiac function is needed; however, the presence of a loss of function mutation could adversely diminish their response to stress [94,95].

An interesting finding was that when myocytes from three patients, each with different truncating BAG3 mutations as described above, were observed with immunofluorescence confocal microscopy, myofibrillar disarray was present. Additionally, there was a decrease and relocation of BAG3 proteins in the sarcomeric Z-disc. Myofibrillar disarray was also seen in mice infected with human immunodeficiency virus (HIV). These mice showed normal contractile function at baseline but decreased contractile function after open heart surgery in comparison to WT mice [96]. However, we have not seen myofibrillar disarray in a group of patients with loss of function BAG3 genetic variants [12], nor have we seen myofibrillary disarray in mice with LV dysfunction and BAG3 haplo-insufficiency [35]. Disarray is difficult to separate pathologically from “contraction band necrosis”, a fixation artifact that occurs when the heart is stopped, either due to disease or in investigational studies in experimental animals, while it is in systole. In laboratory experiments with mice, we can stop the heart in diastole by administering deep anesthesia and then stopping the heart with a large volume of cold hypertonic KCl administered intravenously. In mice studied in this manner we saw no disarray (unpublished data).

### 4.2. Cardiomyocyte Structural Integrity and Protein Quality Control

BAG3 has been found to affect cardiomyocyte contractility in response to β-adrenergic stimulation [97]. In left ventricular (LV) myocytes from adult mice, BAG3 localizes to the sarcolemma and t-tubules and colocalizes with the β1 adrenergic receptor and the L-type Ca^2+^ channel. This differs from the diffuse distribution of BAG3 to the sarcomere of neonatal mouse/rat ventricular myocytes [98]. This difference in localization supports other observations that suggest a change in the functional role of BAG3 at different developmental stages [48]. When BAG3 is knocked down by approximately 50% via injection of adenovirus-short hairpin RNA (Adv-shRNA) BAG3, there is an observable alteration in contractile function. [Ca^2+^]_i_ transient amplitudes and single myocyte contraction amplitudes become significantly reduced in BAG3 knockout myocytes after exposure to isoproterenol, suggesting that BAG3 is involved in amplifying cardiac contraction in response to β-adrenergic stimulation [97]. BAG3, the β1AR, and the L-type Ca^2+^ channel associate with one another in adult cardiac myocytes, likely to form a macromolecular signaling complex, which would also support the development-dependent effects of BAG3 on cardiac contractility. By contrast, BAG3 downregulation prolongs the action potential (AP), regardless of isoproterenol exposure. AP prolongation is associated with increased risk of arrhythmias. Therefore, the effects of decreased BAG3 on AP prolongation adds a further layer of complexity to the clinical presentation of patients with DCM and end-stage heart failure [97].

### 4.3. BAG3 as a Stress-Responsive Protein in the Heart

BAG3 levels can be altered by various mutations in the *BAG3* gene, but even in the absence of a mutation, elevated BAG3 levels have been found to be secreted by cardiomyocytes in response to stress, and also in the serum of patients with heart failure, hypertension, and hypertension with type 2 diabetes mellitus (T2DM) [19]. Additionally, BAG3 expression is increased in response to other stressors, such as heavy metals, high temperatures [99,100], oxidants, proteasome inhibitors [101], light damage (in the retina) [101], and hypoxia [102]. This may be due in part to the fact that these high stress states can cause damage to tissues and proteins, requiring an increase in mechanisms such as apoptosis and autophagy, all of which are mediated in part by BAG3. Stress can also induce heat-shock factor 1 (HSF1) as well as the HSF target gene DNAJB1 in smooth muscle. HSF1 is involved in the upregulation of BAG3 expression in both cardiac and non-cardiac cells [28]. In the heart, HSF-1 expression in the left ventricle and nuclear localization is correlated with LV BAG3 expression. In patients with HF, this HSF-1 pool decreases and could result in the subsequent decrease in BAG3 levels seen in end-stage HF [24]. Additionally, epinephrine exposure has been shown to upregulate BAG3 in cardiomyocytes, but there are likely other mechanisms for stress-induced BAG3 expression that have yet to be fully identified [103].

BAG3 levels are significantly decreased in the hearts of patients in end-stage HF but without known *BAG3* mutations [89]. It remains unclear whether or not this decrease in BAG3 levels is causative of increases in the progression of HF, or if it is a secondary result of the primary disease process. In the context of HF, the observed impairment of myofilament force-generating capacity (F_max_) in mice appears to be a result of reduced BAG3-mediated sarcomere turnover [35]. However, this remains an area of active research in view of the important role that BAG3 plays in the heart.

### 4.4. Role of BAG3 in Skeletal Muscle

Genetic variants in BAG3 can result in myofibrillar myopathies (MFM) that can present as childhood muscular dystrophies with or without peripheral neuropathy and with or without giant axon disease. Selcen first described a group of cases of MFM with peripheral neuropathy, giant axon disease, and a pattern of inheritance consistent with dominant inheritance [81]. That the resulting skeletal myopathies were the result of a single nucleotide polymorphism that substituted a leucine for the proline commonly found at position 209 (P2090L) as described above [81] is important for understanding that BAG3 has important roles in multiple tissues and organs—the skeletal muscle being one of them [81,104]. In muscle, just as in the heart, BAG3 colocalizes with the proteins filamen and actin at the Z-disk, regulates several cellular processes within the muscle including apoptosis (inhibition) cell survival (stimulation), while also stimulating macroautophagy/autophagy and inhibiting proteasomal degradation, and regulating cellular mechano-transduction. The disintegration of the myofiber Z-disk is an early pathological alteration, which is followed by disintegration of the myofibrils, aggregation of degraded filaments into pleomorphic granular or hyaline inclusions, and abnormal expression of multiple Z-disk–related and other proteins in the affected muscle fibers [105].

It is worth mentioning that other hereditary diseases can look very similar to the presentation in children with P209L. For example, Olive et al. [106] reported studies in small cohorts of patients with myotilinopathy—a disease that has been associated with variable syndromes including limb girdle muscular dystrophy type 1a and a subgroup of patients with a myofibrillar myopathy due to mutations in the myotilin gene (MYOT). Unlike BAG3-related (P209L) disease that first penetrates in children, MYOT mutations cause disease onset between the ages of 44 and 77 with initial symptoms of muscle weakness in the lower or upper leg muscles at the time of presentation. However, the disease eventually involves muscle groups throughout the body. Similarly, Nakano reported a group of patients with myofibrillar necrosis, intracytoplasmic deposits, as well as subsarcolemmal-located non-rimmed vacuoles and streaming Z-lines that were associated with myofibrillar myopathy. They were characterized as unique because of abnormal foci of desmin by light and electron microscopy.

Mutations in BAG3 P209L is a causative factor of the most severe form of MFM, with patients presenting symptoms early, at ages 6–8, and rapidly progressing limb and axial muscle weakness into the development of cardiomyopathy, respiratory failure, and/or neuropathy [107]. Specifically, Selcen et al. identified three patients with heterozygous P209L mutation who all presented in childhood with progressive limb weakness, axial muscle weakness, and eventual development of cardiomyopathy and severe respiratory failure in their teens [81].

As described in the section on angiogenesis, BAG3 can be easily administered to the skeletal muscle by direct injection of a viral vector-BAG3 construct. McClung and Kontos reported that coding variants in mice determined their susceptibility to ischemic limb muscle myopathy by directing autophagy [108]. Specifically, they found that a BAG3 variant (Ile81Met) segregated with tissue protection from hind-limb ischemia. The BAG3 Ile81Met variant that provided protection was found in the C57Bl/6 mouse background whereas the BALB/c mice had a greater degree of limb loss. However, administration of an adeno-associated virus-BAG3Ile81 construct restored muscle function and protected the limb from ischemia comparable to that seen in the BAG3 Ile81Met variant found in the C57BL/6 mice. These beneficial effects were attributed to enhanced prevention from ischemic tissue necrosis with the BAG3^Ile81^ genotype as compared with the BAG3^Met81^ genotype.

Taken together, BAG3 is a multi-functional protein involved in many biological processes that support cellular structures, cell function, and cell homeostasis. Thus, we can designate BAG3 as an important target for future therapeutic interventions for cardiomyopathy, various cancers and neurological disorders. Even a single amino acid substitution can lead to severe disease that while usually affecting young adults can also be seen in children and the elderly. That the disease is multifunctional is clearly evident from the many pathways of the cell that are altered by haplo-insufficiency; however, the presence of significant phenotypic differences in siblings with the same variant point to the need to better understand the complex biology of this fascinating protein. With gene therapy for loss of function BAG3 variants on the horizon and CRISPR technology somewhere behind, it is imperative that those studies continue without delay.

## 5. Role of BAG3 in Cancer Biology

### 5.1. Fundamental Observations

The fundamental role of BAG3 in the biology of cancer cells is identical but physiologically opposite to that in the heart. To understand the role of BAG3 in a cancer cell one only has to think backwards. For example, the death of heart cells leads inextricably to damage or is often lethal. Therefore, virtually all species have developed biological processes that provide an important resilience in the pathways critical for normal heart function. These include many of the pathways already described. For example, under homeostatic conditions, autophagy removes misfolded proteins that would eventually inhibit the normal reprocessing of misfolded proteins and other cell debris. For example, after a period of ischemia, cells and tissues undertake efforts to protect themselves by enhancing all-important autophagy pathways so that there is a shift from necrosis to apoptosis during cardiac injury. As we and others have shown, this shift limits the size of a myocardial infarction [99], a robust system for energy production that can change to secondary fuels when needed and a complex array of proteins that protect the electrical system of the heart. Ironically, cancer cells take advantage of the robustness of many of the same pathways to allow for rapid division, resistance to chemotherapy, enhanced local and distant metastasis, inhibition of apoptosis, and enhanced autophagy, which is better for a cancer cell to do in order to protect the expression of a multifunctional protein whose function is weaved through some of the most important activities in a cell. It is not surprising that BAG3 is a target of cancer cells because BAG3 plays a critical role in protecting the heart from damage in response to heat and other signals that are expressed by cancer cells. We have recently reviewed the role of BAG3 in cancer biology in detail [1], but will point out several cogent points here because the contrast is quite remarkable and gives further support to the concept that BAG3 is a quintessential multi-functional protein.

A common feature of many cancer cells is the presence of normal or supra-normal levels of BAG3 [1]. This elevation in BAG3 may arise due to cellular stress and growth factors found in cancerous cells and has been associated with chemoresistance and adverse outcomes in numerous cancer types. In fact, Li et al. were one of the first to validate the role of the Hsp70-BAG3 interaction as a potential therapeutic target in cancer [109]. Increased BAG3 allows for increased coupling with Hsp70 which supports tumor growth, whereas impaired coupling of BAG3-Hsp70 opposes tumor growth [1]. Ammirante et al. noted that the over-expression of BAG3 supported the growth and metastasis of a variety of tumors, including pancreas carcinomas, lymphocytic and myeloblastic leukemias, and thyroid carcinoma through activation [110]. They further showed that downregulation of BAG3 with a BAG3 siRNA increased survival and that the increased survival was mediated by increased NF-kb activation. BAG3 overexpression in human osteosarcoma cells and melanoma cells promotes survival through the NF-kB pathway. Specifically, they showed that BAG3 alters the interaction between HSP70 and IKKγ, increasing availability of IKKγ and protecting it from proteasome-dependent degradation; this, in turn, resulted in increased NF-kB activity and enhanced cell survival [110].

Further evidence for the role of BAG3 in tumor progression comes from studies using inhibitors of the BAG3-Hsp70 interaction. Li and colleagues concluded that JG-98, an allosteric inhibitor of the BAG3-Hsp70 interactome, had antiproliferative activity on cancer cell lines from various origins [109]. Besides the nf-kb pathway, BAG3-Hsp70 interaction is additionally known to regulate metastasis and survival of cancer cells through pathways involving FoxM1 and HuR transcription factors [109]. Li et al. further concluded that treatment with JG-98 in MCF7 breast cancer cells resulted in reduced levels of FoxM1 and HuR, while increasing levels of cell cycle inhibitors, p21 and p27 [109]. Further, YM-1, an inhibitor of the Hsp70-BAG3 interaction, suppressed tumor growth in mice when administered in vivo [111]. YM-1 administration also led to a failure in autophagy in regulating the clearance of cell debris and misfolded proteins.

Increased expression of BAG3 is especially prominent in hematologic malignancies. A secreted form of BAG3 has been identified in studies on pancreatic ductal adenocarcinoma (PDAC), the form of cancer most often associated with BAG3. Secreted BAG3 can bind to a specific receptor, IFITM2, expressed on macrophages, and induce the release of factors that sustain tumor growth and the metastatic process, most notably BAG3. BAG3 neutralization therefore appears to constitute a novel potential strategy in the therapy of PDAC and, possibly, other tumors [80]. In cells from patients with leukemia, downregulation of BAG3 led to a rapid increase in apoptosis and an enhanced response to chemotherapeutic drugs. The enhanced survival appears to be the result of BAG3 binding to hsp70, which weakens the interaction between hsp70 and anti-apoptotic proteins and inhibits delivery to proteasomes. This increases the levels of anti-apoptotic proteins (akt, gamma component of IKK complex, BRAF, MCL-1, BCl-XL) and thus survival of tumor cells. While this mechanism has been shown in various tumor types, it does not apply to glioblastoma cells. In glioblastoma, BAG3 binds bax in the cytosol which prevents bax translocation to the mitochondria and thus inhibiting apoptosis.

Wilms’ tumor 1 (WT1) gene, an oncogene that has been shown to be overexpressed in the wild-type form in the blast and crisis phase of chronic myelogenous leukemia and also acute lymphoblastic and myeloblastic leukemia, binds to the promoter region of BAG3 and acts as a transcriptional activator [112,113]. In leukemias, it has further been shown that elevated levels of WT1 are associated with a poor apoptotic response to chemotherapy [113]. Cesaro concluded that WT1 enhances expression of BAG3, which contributes to the prosurvival role of WT1 in leukemia [112].

### 5.2. Epithelial-Mesenchymal Transition/Stemness

BAG3 also has the ability to enhance epithelial-mesenchymal transition (EMT), which enables cancer cells to invade surrounding tissues [77]. Habata et al. showed that this effect was attributable to the ability of BAG3 to suppresses miR-29b, an inhibitor of matrix metalloproteinase-2 (MMP2) [114]. By enhancing MMP2 expression, cancer cells can more readily invade neighboring tissues. BAG3 binds to phospholipase-C- via the PXXP domain which likely also enhances metastasis [95]. Support for a role for BAG3 in metastasis and local invasion has also come from studies in monkey embryonic fibroblasts that showed that BAG3 deficient cells had a delay in the development of filopodia, the foot-like extensions of the cell membrane involved in cell migration [77]. Lastly, in glioblastoma stem cells having a sphere-like phenotype, BAG3 depletion decreased sphere-forming activity, SOX-2 expression, and the expression of STAT3, a master regulator of stemness [115]. BAG3 depletion was also associated with decreases in the nuclear levels of phosphorylated (activated) STAT3, whereas ectopic STAT3 overexpression normalized BAG3 levels and function in the glioblastoma cells in which BAG3 had been knocked out. Knockdown of BAG3 resulted in recovery of sphere-forming activity in BAG3-knockdown glioblastoma cells. Consistent with the underlying hypothesis of this review, immunoprecipitation and confocal microscopy revealed that BAG3 physically interacts with STAT3 [115].

This ability of BAG3 to modify the phenotype of cancer cells is directly related to maintaining “stemness”, which is defined as the presence of a subpopulation of cells with stem-like properties that facilitate the ability of the cell to undergo self-renewal and to have the potential for differentiation [77]. Recent studies have shown that BAG3 promotes stem-like cells in breast cancer by interacting with the transcript for CXCR4 [116]. Other reports have opined that BAG3 depletion led to decreased sphere forming activity, that was also associated with decreased SOX-2, a transcription factor that maintains self-renewal, and STAT3, a master regulator of stemness, expression [116]. Taken together, these studies strongly support a role for BAG3 in cancer biology although many of the mechanisms responsible for its effects remain to be clarified.

## 6. Conclusions

To summarize, BAG3 is a multi-functional protein involved in many biological processes that support cellular structure, function, and homeostasis. Fundamental processes supported by BAG3 include apoptosis, autophagy, excitation-contraction coupling, maintenance of the mitochondrial membrane potential, movement of normal cells and metastasis and local invasion of malignant cells, and cell–cell communication. In this context, BAG3 could be considered as a super-multi-function protein. The ongoing efforts to better understand the biology of BAG3 is relevant as no treatment exists for any of the diseases that are attributable either directly or indirectly to alterations of BAG3 expression. Genetic variation has recently been shown to cause abnormal BAG3 function based on whole exome and whole genome sequence data from a group of both normal and abnormal human tissues and confirmed by substantive studies in a mouse model of haplo-insufficiency. For reasons that remain obscure, there has been a relative paucity of investigations of BAG3 science. Despite the relatively few laboratory groups focusing their efforts on understanding the pathobiology of BAG3, there has been a culture of collaboration across those groups despite the fact that they work on different continents. It is probably time to create a world-wide BAG3 society to raise research funds and public interest.

## Data Availability

There is no data to share. Investigators should contact the original authors of any clinical studies that are referenced. Similarly, the reader should contact the individual basic science studies that are mentioned for any reagents, clones, or construct.
